# Impact of Underweight, Overweight, and Obesity on Health-Related Quality of Life in Children with Tetralogy of Fallot Variants

**DOI:** 10.1007/s00246-024-03416-w

**Published:** 2024-03-20

**Authors:** Pip Hidestrand, Birgitta Svensson, Pippa Simpson, Petru Liuba, Constance G. Weismann

**Affiliations:** 1 Department of Pediatric Cardiology, Skåne University Hospital, Lund University, Lund, Sweden; 2 Clinical Sciences Lund, Lund University, Lund, Sweden; 3 Division of Quantitative Health Sciences, Department of Pediatrics, Medical College of Wisconsin, Milwaukee, WI USA; 4 Department of Pediatric Cardiology and Pediatric Intensive Care, Ludwig Maximilian University, Munich, Germany

**Keywords:** Health-related quality of life, Congenital heart disease, Overweight, Obesity, Body mass index, Tetralogy of Fallot, Right ventricular outflow tract

## Abstract

Traditional cardiovascular risk factors put patients with congenital heart disease (CHD) at increased risk for cardiovascular morbidity and mortality. The aim of this study was to evaluate whether body mass index (BMI) is associated with health-related quality of life (HRQoL) in patients with variants of Tetralogy of Fallot (TOF). Patients and parents of children with variants of TOF–CHD were asked to fill out the PedsQL 4.0 questionnaire and provide weight and length. Patients were categorized into low, normal, and high BMI percentiles. Other demographic data were obtained from the Swedish national registry for congenital heart disease (SWEDCON). Statistical analyses included non-parametric Mann–Whitney *U* test, Fisher exact, and Chi-square tests. Eighty-five patients were included. Twelve were overweight or obese, 57 had a normal BMI, and 16 were underweight. There was a significant difference in age and gender between the groups. Comparing overweight/obese children to those with normal BMI, physical and social functioning were impaired, while emotional and school function were comparable between the groups. This applied to both child and parental assessment. When comparing underweight to normal weight children, school functioning assessed by the parent was the only domain significantly different from patients with a normal BMI. Children with variants of TOF and overweight/obesity have lower HRQoL, particularly in physical and social functioning, while underweight children may have impaired school functioning. We suggest that preventive measures aimed at maintaining a normal weight should be taken early in life to reduce long-term cardiovascular risk in the CHD population.

## Introduction

Congenital heart defects (CHD) are the most common birth defects affecting about 1% of all newborns [1]. Right ventricular outflow tract obstructive (RVOTO) lesions with a ventricular septal defect encompass all degrees of Tetralogy of Fallot (TOF) ranging from minimal obstruction to pulmonary atresia with major aortopulmonary collaterals or double outlet right ventricle. They constitute about 10–15% of CHD [2, 3].

As treatment options have continuously improved over the last 50 years, most patients reach adulthood [4]. Thus, the focus has been shifting from short-term survival to health-related quality of life (HRQoL), exercise capacity, and long-term survival. Children with TOF have been reported to have comparable HRQoL to healthy children [5]. However, at least 20% of children with right ventricular outflow tract lesions report physical limitations and/or cognitive difficulties [6].

Parallel to the improving survival in CHD, an overweight/obesity epidemic has been developing in the general population [7]. Overweight is defined as having a body mass index (BMI) at or above the 85th percentile for children and at or above 25 kg/m^2^ for adults, as long as criteria for obesity are not met. Obesity is defined as having a BMI at or above the 95th percentile for children and at or above 30 kg/m^2^ for adults. As overweight and obesity are becoming more prevalent in the general population, the prevalence is also increasing in patients with CHD. A recent systematic review article demonstrated that the prevalence of overweight and obesity in children and adults with CHD was similar to the general population and increasing with age. However, in patients with CHD the risk for cardiovascular morbidity, such as coronary artery disease, stroke, and heart failure, appears to be disproportionally increased compared to the general population [8].

Being overweight or obese is also associated negatively with HRQoL in general. Reports have shown that all domains (emotional, social, physical, and school function) of health-related HRQoL are affected in overweight children [9, 10]. Although more research has focused on this area, there still is much to be learned about HRQoL in patients with CHD. There are mixed results with reports of children with CHD, a HRQoL comparable to healthy children and other reports of children with CHD having lower HRQoL scores compared to their healthy peers [5, 11, 12]. In particular, it has not been evaluated to date how weight is related to HRQoL in patients with CHD.

It was the aim of this study to investigate whether there is an association of BMI with HRQoL in patients with variants of TOF. We hypothesized that having an abnormally low or high weight based on BMI percentile is associated with lower HRQoL.

## Methods

The study reported herein is based on data from a previously reported prospective study [6]. For details regarding the methodology, please refer to the original study. Briefly, the patients were obtained from the Swedish national registry for congenital heart disease (SWEDCON) and surgical database. Patients with various forms of TOF ranging from mild right ventricular outflow tract obstruction to pulmonary atresia with major aortopulmonary collaterals were included. Although in the original study, three questionnaires, PedsQL 4.0, PedsQL cardiac module, and DISABKIDS, with instructions were sent out by mail to the children and parents, in the current study we only analyzed the children’s and parents’ response to the general pediatric questionnaire PedsQL4.0 in relation to BMI. The PedsQL 4.0 questionnaire consists of 23 items in four domains: physical functioning (eight items), social functioning (five items), school functioning (five items), and emotional functioning (five items) and include parallel child and parent report. PedsQL 4.0 “may be applicable in clinical trials, research, clinical practice, school health settings and community populations” [13].

Body weight (kg) and height (cm) were part of the questionnaire sent out to parents, but not previously incorporated into the analyses. Other demographic data were obtained from the national registry (SWEDCON). BMI (kg/m^2^) was calculated. We defined being underweight (group Low BMI) as having a BMI <5th percentile, normal weight (group Normal BMI) as having a BMI between the 5th percentile and less than the 85th percentile and being overweight or obese (group High BMI) as having a BMI at or greater than the 85th percentile according to the U.S. Centers for Disease Control and Prevention (CDC). Obesity was not analyzed separately due to the small sample size. In addition, patients were grouped based on underlying diagnosis type into TOF with antegrade pulmonary blood flow and other lesions affecting the right ventricular outflow tract.

Statistical analyses: The PedsQL4.0 total score was the primary outcome along with secondary outcomes of its four domains, and items in the scale were analyzed retrospectively with respect to BMI categories. Categorical variables are presented as *N* (%), continuous variables as median (interquartile range, IQR). Group comparisons were performed using the Fisher exact, Chi-square, and Mann–Whitney *U* test as appropriate. A *p*-value of <0.05 was considered statistically significant. Statistical analyses were performed using Statistical Package for Social Sciences, version 29 (IBM SPSS, Chicago, IL).

## Results

**Fig. 1:**
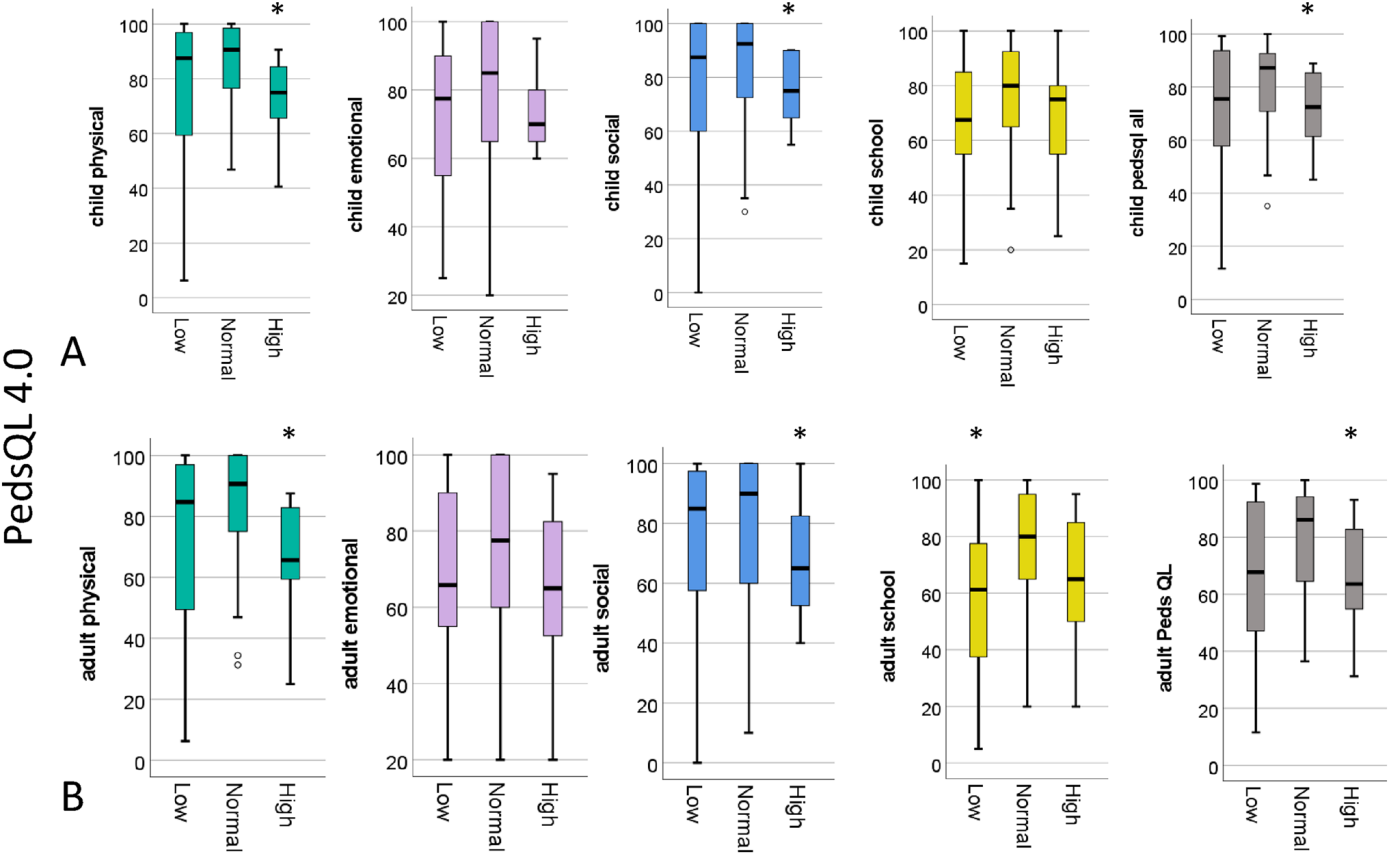
PedsQL4.0 results for the child (**A**) and parent (**B**) comparing groups Low Body Mass Index (BMI), Normal BMI, and High BMI. Median (interquartile range) is displayed. *p values < 0.05 were considered statistically significant

HRQoL data obtained from PedsQL4.0 were available for 97 patients with TOF variants. Of these, height and weight were available for 85 patients with the HRQoL outcome. Six children and four parent questionnaires were not returned. Patients with only one completed form by either the child or the parent were included in the analyses. Across the entire cohort, median age was 12 (IQR 10–15.5) years. Fifty-two (61%) were males. Median BMI was 18.1 (IQR 15.8–21.5) kg/m^2^ and median BMI percentile was 44.1 (17.3–79) %. Regarding the anatomical diagnoses, 59 patients (69.4%) had TOF with antegrade pulmonary blood flow. The remaining 26 patients had TOF with pulmonary atresia with or without major aortopulmonary collaterals (*n* = 14, 16.5%) or double outlet right ventricle with RVOTO (*n* = 12, 14.1%). Median age at first surgery was 3 (IQR 2–3) months and median number of surgeries was 2 (IQR 1–3). Sixteen patients (19%) were underweight (group Low BMI), 57 (67%) were normal weight (group Normal BMI), and 12 (14%) were overweight or obese (group High BMI) (Fig. 1).

Cohort characteristics based on BMI category are shown in Table 1. Groups High BMI and Low BMI were each compared with group Normal BMI. There was no significant difference in age, sex, or length. Naturally, weight, BMI, and BMI percentile were significantly different between the groups. Diagnosis type, presence of a 22q11.2 deletion, age at first surgery, presence of a conduit, and number of surgeries did not differ between the groups (Table 1).

**Table 1:** Cohort characteristics based on BMI category “normal” vs. “high” (BMI percentile ≥ 85%) or “low” (BMI percentile < 5%), respectively

	Variable	Normal BMI (*n* = 57)	High BMI (*n* = 12)	*p*^High vs. Normal^	Low BMI (*n* = 16)	*p*^Low vs. Normal^
General	Age	13 (10–15)	11.5 (9.3–17.5)	0.804	12 (10.3–16.8)	0.997
	Sex (male)	34 (59.6%)	8 (66.7%)	0.753	10 (62.5%)	1
	Length (cm)	156 (140.5–165)	152 (137.3–170.8)	0.848	146 (131.3–158)	0.123
	Weight (kg)	45.0 (34–57.5)	51.5 (39.8–74.5)	0.064	29.5 (24.3–41.5)	0.001
	BMI (kg/m^2^)	18.4 (17.1–21.0)	23.1 (20.6–26.5)	<0.001	14.2 (13.5–16.1)	<0.001*
	BMI percentile (%)	50.4 (26.5–75.4)	91.4 (86.8–94.4)	<0.001	1.6 (0.3–2.9)	<0.001*
Diagnosis	Tetralogy of Fallot	39 (68.4%)	11 (91.7%)	0.157	9 (56.3%)	0.385
	Other RVOT defect	18 (31.6%)	1 (8.3%)		7 (43.8%)	
Genetics	22q11.2del	4 (7%)	1 (8.3%)	1	1 (6.3%)	1
Surgeries	*Age of 1st surgery*			0.188		0.274
	<1 month	16 (28.1%)	0 (0%)		1 (6.3%)	
	1–3 months	9 (15.8%)	3 (25%)		4 (25%)	
	3 months–1 year	26 (45.6%)	8 (66.7%)		8 (50%)	
	>1 year	6 (10.5%)	1 (8.3%)		3 (18.8%)	
	*Conduit*	22 (38.6%)	6 (50%)	0.527	7 (43.8%)	0.776
	*Number of surgeries*			0.385		0.507
	1	24 (42.1%)	4 (33.3%)		5 (31.3%)	
	2	17 (29.8%)	7 (58.3%)		4 (25%)	
	>2	16 (28.1%)	1 (8.3%)		7 (43.8%)	

Data are presented as median (25th–75th percentile) or number (percent) as appropriate

*p values < 0.05 were considered statistically significant

When comparing HRQoL between groups High BMI and Normal BMI using PedsQL4.0, children in the former group had significantly impaired physical and social functioning as well as a lower summative score (Table 2). Emotional and school function, by contrast, were comparable between the groups. This applied to both child and parental assessment.

**Table 2:** PedsQL4.0 results based on BMI category “normal” vs. “high” (BMI percentile ≥ 85%) or “low” (BMI percentile < 5%), respectively

	Normal BMI (*n* = 57)	High BMI (*n* = 12)	*p*^High vs. Normal^	Low BMI (*n* = 16)	*p*^Low vs Normal^
*Child (N)*	56	9		14	
Physical functioning	91 (76–99)	75 (61–86)	0.009*	88 (59–98)	0.237
Emotional functioning	85 (65–100)	70 (65–85)	0.187	78 (55–91)	0.301
Social functioning	93 (71–100)	75 (65–90)	0.031*	88 (60–100)	0.264
School functioning	80 (65–94)	75 (50–82)	0.267	68 (49–89)	0.277
Total PedsQL4.0 Score	87 (70–100)	73 (61–86)	0.046*	75 (57–95)	0.269
*Parent (N)*	54	11		16	
Physical functioning	91 (74–100)	66 (59–84)	0.002*	85 (48–98)	0.170
Emotional functioning	77 (60–100)	65 (50–85)	0.128	66 (55–90)	0.170
Social functioning	90 (60–100)	65 (45–85)	0.034*	85 (50–100)	0.240
School functioning	80 (63–95)	63 (45–90)	0.110	61 (36–81)	0.032*
Total PedsQL4.0 Score	86 (64–94)	63 (49–84)	0.026*	68 (47–94)	0.110

Data are presented as median (25th–75th percentile) or number (percent) as appropriate

*p values < 0.05 were considered statistically significant

Looking at the item level in the affected physical domains, both child and parent of children with High BMI reported “It is hard for me to run” (*p*^child^ = 0.017, *p*^parent^ < 0.001), “It is hard for me to do sports activity or exercise” (*p*^child^ < 0.001, *p*^parent^ = 0.002), “It is hard for me to lift something heavy” (*p*^child^ < 0.021, *p*^parent^ = 0.004), and “I have low energy” (*p*^child^ = 0.028, *p*^parent^ = 0.012). Interestingly, only the parents reported that “It is hard for the child to do chores around the house” (*p*^child^ = 0.308, *p*^parent^ = 0.012). The other questions in the physical domain referring to walking more than a block, taking care of personal hygiene and pain were not significantly different when compared to the Normal BMI group (all *p* = 0.241–1).

In terms of social functioning, High BMI children reported “I have trouble getting along with other kids” (*p*^child^ = 0.009, *p*^parent^ = 0.480), and “I cannot do things that other kids my age can do” (*p*^child^ = 0.036, *p*^parent^ = 0.008). Only the parents reported that “it is hard for the child to keep up when playing with other kids” (*p*^child^ = 0.382, *p*^parent^ = 0.039). “Other kids do not want to be my friend” or “Other kids tease me” did not reach statistical significance in comparison to normal BMI children (*p* 0.182–0.255).

Although school functioning did not differ between the groups overall, children with high BMI, their parents reported that the child has “trouble keeping up with schoolwork.” Absenteeism due to illness though did not appear to be a problem according to the parents (*p* > 0.3).

Next, we compared groups Low BMI and Normal BMI (Table 2). Parents of children with Low BMI, but not the children themselves, reported lower school functioning overall. According to their parents, children in the Low BMI group tended to “forget things” more often (*p*^parent^ = 0.011), and tended to have more “difficulties paying attention in class” (*p*^parent^ = 0.077), “missed school more often because they were not feeling well” (*p*^parent^ = 0.057) or “missed school to go to the doctor” (*p*^parent^ = 0.100), and had “trouble keeping up with schoolwork” (*p*^parent^ = 0.112). None of the other domains were significantly different between groups Low and Normal BMI, though there was a trend towards lower physical functioning as reported by both child and parents and a trend towards a lower summative score reported by the parents (*p*^parent^ = 0.110).

## Discussion

To our knowledge, this is the first paper that has investigated the association between BMI and HRQoL in patients with variants of TOF. We found that patients with a high BMI have impaired physical and social functioning compared to those with a normal BMI. Interestingly, patients with low BMI appear to have preserved functioning in these domains, but parents do report impaired school function, which may be in part due to illness-related school absences.

Overweight and obesity is a highly prevalent problem not only in the general population, but also in patients with CHD. Studies show that the percentage of CHD patients who are overweight or obese is similar to the general population, though some data suggest that patients with severe CHD may be more at risk for being underweight [14–16]. In a recently published systematic review article on the subject, the reported prevalence of overweight in children with CHD was between 9.5% and 31.5%, and the obesity prevalence was between 9.5% and 26% [14]. In adults with congenital heart disease (in ACHD), the prevalence of overweight was between 22% and 53%, and that of obesity between 7% and 26%. The reported prevalence varies widely between the studies, probably due to geographical differences. However, in general the reported prevalence of overweight and obesity was similar to the reference population and increasing with age. Nonetheless, patients with complex CHD have been shown to have poor eating habits and low levels of physical activity [17–21].

Historically, CHD patients were often restricted from physical activity by parents and physicians for fear of an adverse event [22, 23]. Physical activity, among other beneficial aspects, is associated with reduced risk of obesity in CHD though [20]. In addition, multiple studies have shown that for most patients the risk for an adverse event during physical activity is negligible [24]. Current guidelines of the European Society of Cardiology on physical activity in patients with heart disease provide CHD-type and severity-specific recommendations for physical activity [25]. Individual exercise recommendations can and should be based on these guidelines. Thus, in the current era, physicians have changed their practice in how they advise patients in terms of physical activity and possible restrictions. Nowadays, most physicians will encourage physical activity with potentially some recommendations for restrictions based on the type of defect the patient has [20]. TOF patients without residual lesions should typically not be restricted from physical activity. Even those with moderate residual lesions may participate in moderate levels of physical activity [26]. In spite of this, a high percentage of physicians continues to restrict their CHD patients from sports participation [20, 27].

Not only do diet and exercise play a role in CHD patients developing overweight and obesity but there are also multiple psychosocial effects associated with having CHD that can increase the risk of developing obesity. Anxiety, depression, and poor self-esteem are experienced by a significant number of patients with CHD [28, 29]. Chronic stress from dealing with the challenges of having a significant CHD can also occur [30]. These factors are associated with obesity as well [31, 32].

It is well known that overweight or obese children without CHD often have a lower social quality of life [33, 34]. They commonly have negative interactions with other children including teasing or bullying [35]. The question is often asked if overweight children have a lower social quality of life because they are obese or do they have a lower social quality of life that then leads them to behaviors which increase the risk of developing obesity (i.e., not interacting with peers in sports, overeating to counteract negative feelings, etc.). Jackson et al. investigated this interaction and discovered that lower social abilities were associated with development of obesity but being obese did not decrease patients’ social abilities [35]. One could speculate that certain patients with a chronic illness may have a lower social ability due to their chronic illness. This lower social ability then leads to behaviors mentioned above that can increase the risk for obesity.

Otherwise healthy obese children have a lower HRQoL in the physical activity domain than normal weight children [36]. Patients with excess weight find it difficult to be more active due to increased weight and decreased cardiopulmonary fitness [34]. This in turn can lead to a further decrease of physical activity which again can amplify weight gain. In patients with CHD, it is often less clear whether the CHD is the cause of lower physical functioning leading to overweight/obesity or vice versa. Certainly, when looking at HRQoL in adults with CHD, patients with severe CHD such as cyanotic patients have a lower physical HRQoL than patients with less severe defects [37]. This is not surprising as cyanotic patients are impaired physically due to their underlying disease and so will have a decreased physical HRQoL.

Obesity also has an effect on being able to be physically active when taking into account severity of the defect in TOF patients. Aly et al. found that obese patients with “simple” TOF had lower biventricular function and exercise capacity compared to TOF patients with normal BMI [38]. Patients with more complex disease were excluded in this study. Further investigations with larger groups including patients in all parts of the spectrum of this complex disease are needed to answer the question of whether a high BMI contributes to biventricular dysfunction and worse outcome.

There are mixed reports of HRQoL when comparing children with CHD to those without. Uzark et al. reported that children with CHD have a lower HRQoL than children without [12]. Moreover, patients with more complex CHD have reported having a lower HRQoL compared to children with simple congenital heart lesions [39]. Reasons for this include but are not limited to requiring surgery, taking medications, and having more symptoms/limitations when compared to children with simple heart lesions. Others report that children with CHD and TOF, in particular, have similar HRQoL measures compared to their healthy peers. Of particular interest is that HRQoL appears to correlate positively with the child’s ability to exercise [5].

Obese patients with TOF variants examined in this paper have two risk factors (obesity and chronic medical condition) for a decreased HRQoL compared to children without either. New recommendations are available regarding exercise restrictions related to the patient’s specific heart lesion [25, 40]. Providers should strongly encourage a healthy lifestyle with a well-balanced diet and physical activity to help patients minimize the risk of becoming overweight or obese. It is known that being overweight or obese increases cardiovascular morbidity and mortality, which CHD patients are already at increased risk for—even in the absence of additional risk factors [8]. Discussing with children periodically HRQoL issues may detect potential problems early and help implement effective strategies to increase HRQoL and help mitigate some of the risk factors for developing obesity. We suggest that patients with CHD and overweight should be referred to outpatient rehabilitation programs including physical activity and nutrition counseling early, i.e., before obesity occurs.

## Limitations

An existing HRQoL data set of children with TOF variants was used. Due to missing data on height and/or weight, 12 patients had to be excluded. This was data collection from one country with including only patients with TOF variants, thus generalizability to other heart defects is limited. Larger studies would need to be done for better generalizability. This was a small study with relatively few overweight or obese patients (14%). Given the small population size, it was not possible to analyze overweight and obesity separately. The surveys were filled out at home, and in a few cases, as described above, only the child or the parent returned the completed questionnaire. There may be some selection bias given that the patients completing the questionnaire may be biased. In addition, we did not have detailed clinical information or echocardiographic data available. However, hemodynamically relevant residual defects are usually corrected early in life while the median age of our cohort was 12 years. Lastly, we did not take into account variables such as socioeconomic status which may also impact HRQoL.

## Conclusions

This paper demonstrates an association between overweight or obese children with RVOTO and decreased HRQoL, particularly in social and physical functioning. As being overweight or obese adversely effects cardiovascular health in patients with CHD, the importance of preventative measures to maintain a normal weight cannot be overemphasized.
